# Immunogenicity of Pfizer mRNA COVID-19 Vaccination Followed by J&J Adenovirus COVID-19 Vaccination in Two Patients with Chronic Lymphocytic Leukemia

**DOI:** 10.1155/2022/6831640

**Published:** 2022-02-04

**Authors:** Zoe L. Lyski, Myung Sun Kim, David Xthona Lee, David Sampson, Hans-Peter Raué, Vikram Raghunathan, Debbie Ryan, Amanda E. Brunton, Mark K. Slifka, William B. Messer, Stephen E. Spurgeon

**Affiliations:** ^1^Department of Molecular Microbiology & Immunology, Oregon Health & Science University (OHSU), Portland, OR 97239, USA; ^2^Knight Cancer Institute, Oregon Health & Science University (OHSU), Portland, OR 97239, USA; ^3^Division of Neuroscience, Oregon National Primate Research Center, Oregon Health & Science University, Beaverton, OR 97239, USA; ^4^OHSU-PSU School of Public Health, Portland, OR 97239, USA; ^5^Department of Medicine, Division of Infectious Diseases, Oregon Health & Science University (OHSU), Portland, OR 97239, USA

## Abstract

Individuals with chronic lymphocytic leukemia (CLL) have significant immune disfunction, often further disrupted by treatment. While currently available COVID-19 vaccinations are highly effective in immunocompetent individuals, they are often poorly immunogenic in CLL patients. It is important to understand the role a heterologous boost would have in patients who did not respond to the initial two-dose mRNA vaccine series. SARS-CoV-2 specific immune responses, including antibodies and memory B-cells, CD4 and CD8 T-cells were assessed prior to vaccination, as well as postinitial vaccination series and post-third dose in two subjects. One subject seroconverted, had RBD-specific memory B-cells and spike-specific CD4 T-cells while the other did not. Both subjects had a spike-specific CD8 T-cell response after the original mRNA vaccination series that was further boosted after the third dose or remained stable. The results of this study, however small, are especially promising to CLL individuals who did not seroconvert following the initial mRNA vaccination series.

## 1. Introduction

Chronic lymphocytic leukemia (CLL) is characterized by the monoclonal proliferation of dysfunctional B-cells, leading to a broad range of immune defects. CLL patients face significant risk of morbidity and mortality from infections [1], including from SARS-CoV-2, the causative agent of COVID-19 [2]. Vaccines can be instrumental in mitigating the risk of infections in CLL; however, responses to vaccination are highly variable and significantly influenced by CLL disease status, baseline characteristics, types of vaccine, and active CLL therapy [3].

Although current COVID-19 vaccines elicit robust immunity in immunocompetent hosts [4], the antibody response in CLL patients is highly variable [5–7] and particularly poor in patients with low total immunoglobulin levels, those that have had anti-CD20 monoclonal antibodies within the past year, or those that are undergoing active therapy with agents such as Bruton's tyrosine kinase inhibitors (BTKi). The best responses to date have been in CLL patients who are in remission and/or years out from active treatment.

Given decreased vaccine efficacy in CLL, an additional dose of vaccine may be beneficial in CLL patients, especially given the rise of variants of concern (VoCs). Initial data from solid organ transplant recipients on immunosuppression as well as individuals with solid tumors on active therapy showed a role for additional vaccination [8, 9]. This led to the FDA extending the EUA for Pfizer-BioNTech and Moderna mRNA vaccines to include additional doses in immunocompromised patients. However, these results may not be generalizable to CLL, and additional studies are needed to better define vaccine responses in the CLL patient population, including the role of mixing mRNA vaccination with other vaccine formulations, such as the adenovirus vectored vaccine Ad26COV2.s, commonly known as the Johnson and Johnson (J&J) vaccine.

## 2. Case Report

Here we describe two CLL patients who “self-referred” to outside pharmacies for an additional vaccination with the J&J COVID-19 vaccine following 2 doses of the BNT162b2 vaccine (Pfizer-BioNTech). Both patients had previously been enrolled as study subjects in an IRB-approved observational study (OHSU IRB# 21230) to investigate immune response following COVID-19 vaccination. The additional J&J dose was subsequently self-reported to the study team. On initial enrollment, demographics, CLL disease characteristics, and treatment details were collected (Table 1), and baseline laboratory values were obtained, including semiquantitative SARS-CoV-2 spike antibody titer, serum IgG, a complete blood count, and multicolor flow cytometry measuring immune cell populations (Table 1). Whole blood was collected for additional serologic and cellular studies.

SARS-CoV-2 spike receptor binding domain (RBD)-specific antibody levels were tested by ELISA and endpoint titers were calculated as previously described [10]. In addition, baseline PBMC samples were functionally tested for the presence of SARS-CoV-2 spike RBD-specific memory B-cells (MBCs) by limiting dilution assay as previously described [11, 12]. Briefly, PBMCs were serially diluted and incubated with a stimulation cocktail in which the MBCs present within PBMCs differentiated and became antibody-secreting cells. The supernatants were collected 7 days later and assayed for antigen specificity by RBD-ELISA. This allows one to functionally test MBC-derived antibodies and back-calculate the frequency of total IgG-secreting MBCs as well as RBD-specific MBCs [11, 12]. In addition, CD4+ and CD8+ T-cells were also functionally assessed for the presence of IFNʹ*γ* and TNF*α* secretion following spike protein-derived peptide stimulation. Briefly, PBMCs were stimulated with 2 peptide pools of overlapping (10AA) 17mers representing the SARS-CoV-2 spike protein (BEI Resources). Following stimulation, the cells were stained as previously described [13, 14]. Data was acquired on an LSR Fortessa (Becton Dickenson) and analyzed using FlowJo software. Cytokine expression in medium-alone cultures was subtracted from peptide-stimulated cultures to calculate peptide-specific cytokine expression. Responses to both peptide pools were added together to yield the total frequency of SARS-CoV-2-specific cytokine-producing CD4+ and CD8+ T cells.

**Figure 1:**
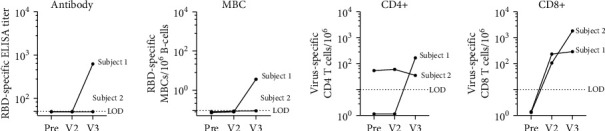
Immune response to COVID-19 vaccination in CLL subjects. RBD-specific antibody titer. Subjects without a detectable antibody titer (<1 : 50 serum dilution) were assigned a value of 49. The limit of detection (LOD) is 50. Frequency of RBD-specific MBCs per 10^6^ CD19+ B-cells following ex vivo stimulation. Subjects who did not have a detectable response were assigned a value between 0.07 and 0.09. The limit of detection (LOD) is 0.1. SARS-CoV-2 spike peptide-reactive CD4 and CD8 T-cells are defined as double positive for IFN*γ* and TNF*α* cytokine secretion. Patients who did not have a detectable T-cell response were assigned an arbitrary number less than 2. The limit of detection (LOD) is 10. Visit 1 (pre) blood draw was taken 21 and 40 days prior to the Pfizer vaccine series (2-doses). Visit 2 (V2) blood draw was taken 33 and 24 days post vaccination, and Visit 3 (V3) was drawn 30 and 27 days after the 3rd vaccination with J&J.

Neither subject had prevaccination B-cell responses as measured by RBD-specific antibodies or MBCs. Neither had a virus-specific CD8+ response at baseline. While Subject 2 had spike peptide-reactive CD4+ T-cells at baseline, these cells were unresponsive and did not expand following vaccination. In contrast, CD8+ responses were observed after mRNA vaccination in both subjects (Figure 1). It has previously been reported that SARS-CoV-2 naïve individuals may have preexisting cross-reactivity to SARS-CoV-2 peptides through prior infection by common cold coronaviruses: SARS-CoV-2 specific CD4+ T-cells have been identified in 20–50% of people without SARS-CoV-2 exposure or vaccination [15].

Approximately four weeks after initial vaccination, neither subject had detectable RBD-specific SARS-CoV-2 antibodies or MBCs. Both had measurable vaccine-induced CD8+ T-cell responses following mRNA vaccination, although CD4+ responses did not appear to increase above baseline (Figure 1).

Subject 1 received the J&J vaccine 104 days and Subject 2 received 81 days after completion of the BNT162b2 vaccine series. Following J&J vaccination, additional samples were obtained from Subject 1, 30 days after the third vaccine, and Subject 2, 27 days following the third vaccine. Interestingly, Subject 1 had undetectable RBD-specific antibodies, RBD-specific MBCs, and virus-specific CD4+ T-cells after the initial vaccination series. However, following an additional vaccination, all three measures increased above the limit of detection: RBD-ELISA titer of 625, RBD-specific MBC frequency of 3.6/10^6^ B-cells, and 166 spike-specific CD4+ T-cells/10^6^, and a spike-specific CD8+ T-cell response that remained stable and did not boost appreciably following the 3^rd^ vaccination (Figure 1). Subject 2 did not seroconvert or have detectable virus-specific MBCs after their primary mRNA vaccination series; however, they had a spike-specific CD8+ T-cell response that was further boosted after a 3^rd^ dose and a virus-specific CD4+ response that did not change following the original vaccine series or the 3^rd^ dose of J&J.

## 3. Discussion

Other than subject age (60s vs 80s), the most notable difference between the subjects' baseline characteristics (Table 1) is that Subject 1 was treatment naive, while Subject 2 had undergone previous treatment (6 years ago) with obinutuzumab, an anti-CD20 mAb, and is currently on active treatment with ibrutinib since 2017. Both had baseline B-cell frequencies outside of the normal range, with Subject 1 exhibiting a low percentage of naïve B-cells (0.092) and a high percentage of MBCs (59.1), while Subject 2 had a low percentage of naïve B-cells (11.37) and MBCs (0.45). Although Subject 2 had mild hypogammaglobulinemia, neither had a history of recurring infections or a need for IgG supplementation. Levels of baseline CD4+ and CD8+ T-cells (absolute values) were also normal in each subject prior to vaccination (data not shown). Both had very low percentages of naïve B cells which could explain the initial poor response to vaccination. The significance of the increased percentage of MBCs in Subject 1 is unclear, but does suggest some broader preservation of normal B cell maturation and immune function. Although Subject 1 did have an immune response, antibody levels were relatively low as compared to some of the levels observed in immunocompetent postvaccine populations [16] and certain CLL populations [4]. The clinical significance of specific antibody levels remains unknown.

Active treatment with Bruton's tyrosine kinase (BTK) inhibitors like ibrutinib may have a profound impact on B-cell survival, differentiation, and production of antibodies as the absence of intact BTK-dependent B-cell receptor mediated signaling prevents B-cells from differentiating into mature peripheral B-cells. Immune responses following vaccination or natural infection are limited in these patients [17]. Recall to antigens encountered prior to treatment appears to remain largely intact; however, response to novel antigens encountered during treatment seems to be abrogated. Subject 2 has been on ibrutinib for over four years. The impact of prolonged treatment vs. shorter-term BTK inhibition on immune responses is unknown. However, clinical data [18] suggest some improvement in humoral immunity with prolonged (>6 months) treatment. T-cells are also disrupted in individuals with CLL and even further disrupted with BTK treatment [19]. In the cases presented here, both subjects did have an increase in virus-specific CD8+ T-cells; however, the significance is unclear in terms of protection, as neutralizing antibodies are often viewed as the correlate of protection against COVID-19.

## 4. Conclusion

The results of this study, however small, provide initial evidence that a 3^rd^ vaccination against COVID-19 with the heterotypic vaccine Ad26COV2.s results in an immune response that was not observed following the recommended 2-dose mRNA vaccination series. This is especially promising news to subjects who are treatment naïve, not currently in active treatment, or who may consider vaccination before beginning active treatment.

## Figures and Tables

**Table 1:** Baseline characteristics and demographics for subjects included in the study.

Subject ID	Age	Gender	Date of diagnosis	Current treatment	Prior treatment	IgG mg/dL	Absolute lymphocyte Count (K/mm3)	B-cells (CD19+) %	Naïve B-cells (IgD+CD27−) %	Memory B-cells (IgD−CD27+) %	B1 B-cells (CD5+CD19+) %
						(768–1632)	(1.00–4.80)	(4–17)	(50–80)	(5–21)	(<6)

1	60's	F	17/02/2014	None	None	834	21.09	76	0.092	59.1	76.18
2	80's	F	02/01/2014	Ibrutinib (2017)	Obinutuzumab × 6 cycles (completed 2015)	510	5.93	61	11.37	0.45	59.96

*Note.* Normal ranges for each of the B-cell subsets are in parenthesis under each B-cell type.

## Data Availability

The data used to support the findings of this study are included within the article.
